# Teachers' perspectives on physically active learning and student wellbeing in the Nova Scotia Active Smarter Kids Project

**DOI:** 10.3389/fspor.2026.1914074

**Published:** 2026-10-14

**Authors:** Julie E. Campbell, Sarah A. Moore, Son Truong, Daniel G. Rainham, Michelle R. Stone

**Affiliations:** 1 School of Health and Human Performance, Dalhousie University, Halifax, NS, Canada; 2 Pediatric Rehabilitation, IWK Health Centre, Halifax, NS, Canada; 3 Department of Pediatrics, Faculty of Medicine, Dalhousie University, Halifax, NS, Canada

**Keywords:** children, health-promoting schools, physically active learning, teachers, wellbeing, youth

## Abstract

**Introduction:**

Active Smarter Kids (ASK) is a Norwegian physically active learning (PAL) program that integrates physical activity into school lessons for students to learn while moving. In 2017, government departments in Nova Scotia (NS), Canada, partnered with ASK to adapt and implement it locally as the NS ASK Project. Since 2018, teachers in over 130 NS schools have been trained in ASK. International studies and a pilot report from NS have noted several benefits of ASK, such as increased physically active time, academic achievement, and executive function. However, a more comprehensive study of the NS ASK project has not been completed. This paper explored elementary and middle school teachers' perceptions of students' wellbeing and the implementation challenges from a PAL project from NS, Canada.

**Methods:**

Ten teachers (5 elementary, 5 middle school) participated in semi-structured interviews addressing their experience conducting ASK lessons, perceived contributions to students' wellbeing, and any implementation challenges they've encountered. Data were analyzed using reflexive thematic analysis.

**Results:**

Seven themes were developed: 1) Fostering belonging and relationship-building; 2) Opportunities for, and experiencing success in, physical activity; 3) The joy of learning with movement; 4) Sharpening engagement and focus; 5) Pedagogical approaches as catalysts for wellbeing; 6) ‘Running' out of resources; and 7) Navigating the status quo.

**Discussion:**

The project was perceived to support many facets of students' wellbeing through its flexible, adaptable approach, enabling the meeting of different needs and abilities, and positioning it as a promising health-promoting school initiative aligned with global wellbeing frameworks. Implementation challenges, such as a lack of space and curriculum and assessment pressures, can inform efforts to rethink educational systems of similar contexts to remove barriers for PAL.

## 1 Introduction

Many countries around the world have documented that most of their children and youth are not getting enough physical activity (PA) to support their wellbeing (i.e., their physical, cognitive, social, and/or emotional state) (1). For instance, in Canada, the 2026 ParticipACTION Progress Report identified that only 37% of 5–17-year-olds achieved the daily guideline of moderate-vigorous PA (MVPA) (2). Broader concerns around child and youth wellbeing are also emerging, where self-rated physical and mental health has declined in recent years, with just over 50% of youth rating their health as very good or excellent (3). Incidence of ADHD and related learning challenges among Canadian children is also on the rise (4, 5). Together, these trends highlight significant concerns and underscore the importance of supportive environments that foster overall wellbeing.

Schools play a critical role in supporting children's health and wellbeing (6, 7). However, in many countries, a growing focus on academic performance and standards-driven curricula has reduced the time allocated for school-based PA and contributed to increased sedentary behaviour (8, 9). Over time, shifts in school priorities have emphasized outcomes-based academic testing and rigid, “one-size-fits-all” approaches that prioritize standardized assessments while cutting back on “extras” such as recess, physical education (PE), and outdoor learning (10, 11). Physically active learning (PAL), a pedagogical approach that integrates bodily movement into curriculum instruction, has been proposed as a way to counter these trends and promote student wellbeing without reducing academic learning time (12).

Evidence from studies conducted around the world suggests that PAL can promote school-based PA and support students' cognitive and behavioural outcomes such as executive functioning and on-task behaviour (13–15). However, much of this work has been conducted with preschool and elementary-aged students (3–12 years old) (13, 15–17), with limited insight into older grades. Understanding PAL's potential among both older elementary and middle school students (i.e., ∼9–14 years old) is particularly important given the well-documented decline in PA, especially in girls, that begins at the onset of puberty due to significant physical, social, and motivational changes (18, 19). These transitions may alter how students respond to and engage with active learning opportunities. Further, a recent ParticipACTION report has noted that youth (aged 12–17) are the least active age group in Canada (2). Such declining trends in youth's PA point to the crucial need to investigate whether school-based PAL can serve as a sustainable strategy to support activity levels and wellbeing among both children and youth.

Few international and Canadian studies have explored teachers' perceptions of PAL's contribution to students' psychosocial wellbeing and have generally involved small samples of teachers with a focus on pre-designed, classroom-based PAL programs, rather than flexible or typically outdoor-based programs (20–22). In Nova Scotia, a province in eastern Canada with ∼1 million residents, schools have embraced a flexible and typically outdoor-oriented PAL program through the Nova Scotia Active Smarter Kids (NS ASK) project, an adaptation of Norway's ASK study, developed in partnership with Norwegian researchers (23–25). NS ASK provides free professional development sessions to teachers on integrating movement into curricular lessons, both outdoors and indoors, along with implementation support and a database of lesson plan examples (24). Lessons are adaptable for most subjects, designed to be non-competitive, and often involve group-based, simple, active formats such as relay-style tasks, word and number studies, or true/false activities using basic equipment like numbered/lettered bean bags or tiles, large dice, hula hoops, and whiteboards.

NS ASK aligns with broader provincial and global frameworks promoting active and outdoor learning, as well as Universal Design for Learning (UDL) and Health Promoting Schools (HPS) (26–28). Despite strong policy alignment, there have been no comprehensive studies of ASK outside Norway. Understanding how NS ASK operates, including its implementation challenges, in Nova Scotia's educational and cultural context, where PAL is less common than in countries like Norway and Denmark (16, 29), is critical for supporting its sustainability and adoption in similar educational contexts.

As the primary implementers of PAL/NS ASK and those who know their students best, teachers offer valuable insights into the impacts of these lessons on student wellbeing (30). Teachers make key decisions about whether to use PAL, how often to use it, what subjects to integrate, where lessons take place, and how they are designed. While over 3000 teachers have been trained in NS ASK, not all choose to adopt the practice. Understanding the perspectives of those who have integrated PAL into their teaching is critical for identifying benefits, challenges, and strategies for wider, sustained implementation.

Beyond program-specific evidence, there remains a broader need to understand how PAL supports students' wellbeing, encompassing physical, cognitive, social, and emotional/mental domains, essential for designing PAL approaches that are engaging, sustainable, and meaningful across contexts (31). This comprehensive view aligns with international frameworks such as the United Nations International Children's Emergency Fund (UNICEF) (32) dimensions of child wellbeing and the World Health Organization (WHO)/United Nations Educational, Scientific and Cultural Organization's (UNESCO) HPS domains of adolescent wellbeing (28). To explain why and how PAL may enhance wellbeing, Self-Determination Theory (SDT) (33) offers a valuable lens, positing that wellbeing depends on the satisfaction of three basic psychological needs: autonomy (the experience of choice and volition in one's actions), competence (feeling capable and effective in achieving desired outcomes), and relatedness (feeling connected to and valued by others) (33). Given PAL's frequent outdoor setting, nature relatedness (a sense of connection to nature) can also be considered a complementary psychological need (34). When these needs are met, students are more likely to experience intrinsic motivation, engagement, and positive wellbeing across all domains. Table 1 provides an overview of how the different wellbeing domains connect with established wellbeing frameworks and the three psychological needs of SDT and nature relatedness.

**Table 1:** An overview of how wellbeing domains explored in this study align with those of established wellbeing frameworks and the three basic psychological needs of Self-Determination Theory (SDT) and nature relatedness.

Wellbeing Domain	UNICEF Innocenti Child Wellbeing Dimensions (32)	UNESCO/WHO HPS Adolescent Wellbeing Domains (28)	Connection to SDT Basic Needs (33) and Nature Relatedness (34)
Physical Wellbeing	Physical health	Good health	Competence, Autonomy, Relatedness, Nature Relatedness
Cognitive Wellbeing	Academic proficiency	Learning, competence, education, skills, and employability; Agency and resilience	Competence, Autonomy, Relatedness, Nature Relatedness
Social Wellbeing	Social skills	Connectedness, positive values, and contribution to society; Safety and a supportive environment	Competence, Autonomy, Relatedness
Emotional Wellbeing	Mental wellbeing	Agency and resilience; Safety and a supportive environment	Competence, Autonomy, Relatedness, Nature Relatedness

Since 2018, teachers in over 130 schools of all ages across all Regional Centres for Education (RCEs) (35) have been trained in NS ASK. Early evaluations from the NS ASK pilot study identified several perceived benefits, including an increase in physically active time, student engagement in learning, and motivation to attend school (23). However, this evaluation involved only three grade five classes from a single RCE, and the participating teachers received additional year-long support from the NS ASK team, which was not a part of the broader NS ASK project. Given this context, there is a need to build on its initial evaluation to understand how teachers across different grade levels, regions, and experiences implement and perceive NS ASK for student wellbeing. Additionally, most studies have focused on PA or cognitive outcomes, rather than taking a multi-dimensional view that includes social, emotional, and mental wellbeing dimensions. Therefore, this study aimed to examine elementary and middle school teachers' perceptions of the NS ASK project in relation to students' wellbeing and challenges in implementing it.

## 2 Methods

### 2.1 Study design

This qualitative study was grounded in a constructivist worldview, acknowledging the multiple, socially constructed realities (36). A qualitative description design was used to provide a rich yet straightforward account of teachers' perspectives and experiences with NS ASK (37). Qualitative description was applied through semi-structured interviews with elementary [grades 4–5 (ages 9–11)] and middle school [grades 7–8 (ages 12–14)] teachers who received training in and reported to be currently and regularly conducting NS ASK lessons. Grade 6 was excluded due to inconsistent categorization across schools (sometimes elementary, sometimes middle). The logistics of conducting the research and developing the semi-structured interview guide were co-developed with the research team and a steering committee of administrators, teachers, health promotion specialists, and NS ASK facilitators through three online, collaborative meetings. Incorporating these perspectives ensured the design was practical and relevant, centering the voices of those who deliver NS ASK lessons in schools. This study was granted ethics approval from Dalhousie University's Research Ethics Board (#2023-6947) and from all RCEs. All participants provided online informed consent before participating in interviews.

### 2.2 Positionality

The lead author identifies as a white, able-bodied woman with PhD-level training in child development, movement behaviours, and health promotion, and has experience conducting qualitative data collection and analysis. Prior to data collection, she completed NS ASK facilitator training, which provided familiarity with the program and underlying knowledge and support for NS ASK that could have shaped interpretations. Because of this connection, reflexivity about her positionality remained critical throughout the study.

### 2.3 Participants

Teachers were recruited using purposive sampling to capture a range of implementation experiences across school and teacher characteristics. Recruitment occurred on a rolling basis from April to November 2024 by contacting school principals by email whose school staff had received NS ASK training and had eligible grades. We began by recruiting schools known to the steering committee to have teachers implementing NS ASK lessons and then expanded purposively to reflect variation in school location (RCE and urban/rural setting), school size (student population), teaching experience, length of involvement with NS ASK, and grade level (grades 4–5 and 7–8), aiming for 1–2 teachers per RCE to maximize diversity across the province's seven RCEs, each with distinct NS ASK training and facilitation teams. The research team asked principals to refer them to interested teachers in their school. These teachers then responded by email and confirmed eligibility by explaining if they currently and regularly deliver NS ASK lessons. All participants were required to have sufficient English fluency to participate without a translator.

### 2.4 Semi-structured interviews

The interview guide included five sections (see Supplementary File 1), including school and class background, NS ASK experience (including how often, when, where, and how they conducted NS ASK lessons), perceived contributions to students' social, emotional/mental, and cognitive wellbeing, perceived contributions to students' physical wellbeing, and lastly, challenges associated with NS ASK implementation. Before interviews, teachers completed a brief demographic questionnaire about themselves and their teaching and NS ASK experience. Each teacher then participated in the same 30–60-minute interview conducted by the lead researcher either virtually or in-person at their school. Interviews were audio recorded using a handheld recorder and Microsoft Teams, with Teams' transcription feature verified against the audio files for accuracy.

### 2.5 Data analysis

Qualitative data from teacher interviews were transcribed verbatim and analyzed using inductive, experiential reflexive thematic analysis (RTA) (38). The lead author conducted all coding and led the analytic process, following the non-linear, six-step process of RTA, including familiarization, coding, theme generation, development, review, and refining (38). Familiarization included listening to, reading, and re-reading transcripts, and taking detailed notes in the reflexive journal. Transcripts were then coded inductively, guided by the research questions, using NVivo 14 software (39). Initial themes were generated through visual mapping to cluster similar codes and ideas. Themes were iteratively refined and finalized through discussions with co-authors. These meetings focused on interrogating the coherence of themes, clarifying central organizing concepts, and considering alternative interpretations, including whether additional or more nuanced themes (e.g., pedagogical approaches) were warranted (38). Although no major disagreements arose, co-authors' feedback prompted several refinements to theme boundaries and naming. Reflexive journaling and notetaking and evolving visual maps and code tables were kept to document the analytic process and theme refinement. Consistent with RTA, saturation was not used, but adequacy and richness of the data were assessed through the extent to which the data provided sufficient depth and variation to address the research questions (38).

To support reflexivity, the lead author documented personal reflections, including emerging patterns, potential biases, or assumptions in a reflexive journal after each interview. She disclosed her prior involvement with NS ASK to participants but emphasized that the study aimed to understand all experiences, positive, negative, or mixed, and that the analysis was conducted independently from the NS ASK Project. The interview guide included prompts about challenges and less favourable experiences to reduce the risk of only eliciting supportive narratives. Regular discussions with co-authors with content and methodological expertise, along with attention to negative findings, supported ongoing triangulation and critical reflection to ensure the analysis remained grounded in participants' experiences while recognizing the influence of the researcher's positionality, strengthening the credibility and trustworthiness of the findings (40). The reporting of our qualitative methods was compliant with the Standards for Reporting Qualitative Research (SRQR) guidelines (41) (see Supplementary File 2).

## 3 Findings

### 3.1 Participants' characteristics

In total, fifty-six schools with eligible grades and purposive sampling criteria were contacted to identify participating teachers. Twenty-five schools did not respond; eight had interested teachers but none teaching grades 4–5 or 7–8; and thirteen declined or reported that teachers were too busy at the time. This resulted in a final sample of 10 teachers who participated in the study, where all but one participated through online interviews, and the interviews lasted an average of 46 minutes. Participants all identified as White European and the majority as women (80%). Participants were evenly split between grades 4–5 and 7–8, with teaching experience ranging from 3 to 33 years and varied involvement with NS ASK (1–>5 years). Half held qualifications in PE, health, or PA. Teachers represented nine schools (two taught at the same school) across five of Nova Scotia's seven RCEs, with varied grade configurations and student enrollment (150–650 students). Most schools were in small population centres (66.7%), with fewer in large urban (22.2%) or rural areas (11.1%) (42). See Table 2 for complete teacher and school characteristics.

**Table 2:** Teacher and school characteristics.

Teacher characteristics (*n* = 10)	*N* (%)
Gender	
Woman	8 (80)
Man	2 (20)
Age	
< 35 years	2 (20)
36–50 years	5 (50)
51–65 years	3 (30)
Ethnicity	
White European descent	10 (100)
Identify as having a disability	
No	9 (90)
Prefer not to say	1 (10)
Grades taught	
Grades 4–5	5 (50)
Grades 7–8	5 (50)
Years teaching total, Median (range)	18 (3–33)
Years teaching at current school, Median (range)	7 (2–19)
Teaching qualification in PE, Health, other PA	
Yes	5 (50)
No	5 (50)
Familiar with PAL before NS ASK?	
Yes	0 (0)
No	10 (100)
Number of days per week a NS ASK lesson is delivered	
< 1 day	2 (20)
1–2 days	4 (40)
3 days	4 (40)

School characteristics (*n* = 9)	
School grade compositions	
Includes pre-primary–6 only	3 (33)
Includes grades 7+	6 (67)
Number of students in school	
150–250	3 (33)
251–350	3 (33)
450–650	3 (33)
Location	
Large urban population centre (≥100,000 persons)	2 (22)
Small population centre (1,000–29,999 persons)	6 (67)
Rural (<1,000 persons)	1 (11)
Number of years since initial NS ASK training	
> 5	4 (44)
3–5	3 (33)
1–2	2 (22)

NS ASK, Nova Scotia Active Smarter Kids; PA, physical activity; PAL, physically active learning; PE, physical education.

### 3.2 Themes

Seven themes were developed to represent teachers' perspectives of students' wellbeing and challenges to implementing NS ASK, expanded upon in the following sections. See Supplementary File 3 for a complete theme summary table with related codes and descriptions of codes, representative quotes, and their connections to wellbeing domains.

#### 3.2.1 Theme 1: fostering relationship-building and belonging

Teachers perceived NS ASK lessons as opportunities for students to socialize in their often-collaborative formats, forming new relationships and connections with other students and their teacher, and fostering feelings of belonging and leadership, whereas some teachers also perceived some negative social impacts. NS ASK lessons were perceived to strengthen existing friendships or spark new ones. As one middle school (MS) teacher described:

They start talking about the math and a couple minutes later… they're talking about what they're doing on the weekend [and] starting to form those relationships. (T9, MS)

Teachers also discussed how NS ASK created natural opportunities for teachers and students to connect through shared participation and informal interactions that do not often naturally occur in the classroom.

Students perceived to be on the margins (due to learning needs, social differences, or language barriers) also became more visible and engaged through adapting the lessons to fit their needs. One MS teacher explained:

There's a lot of language barriers [in my school], so when I make up [a NS ASK lesson] …I'll put the question just in their language. So they'll feel more involved… and everybody has to rally around them. (T3, MS)

NS ASK lessons were also seen to encourage leadership amongst students who either thrive academically or physically, as one teacher described: “*[students who excel academically] feel good about themselves because they're kind of leading the jock kids… they've really helped their group because they understood the assignment.” (T3, MS)* Likewise, the students who do not feel as competent academically were still perceived to experience success and belonging by engaging in the lesson physically and helping their team in that way.

Lessons also helped students understand their abilities, strengths, limitations, and how to collaborate effectively, where another teacher discussed the character building involved with collaboration:

Most importantly, it's about them being able to figure out their strengths but also know what their limits are and who they can work with… It's not about [the specific lesson] …it is ‘what have I learned about myself? ‘What do I bring to the team?’ (T8, MS)

At times, some teachers expressed that collaborative tasks lead to one or two students doing all of the team's work, for instance, one elementary school (ES) teacher mentioned: “*You always have the ones that kind of take the lead, and then you have the others that kind of stand back and let the other people do it.” (T2, ES)* This results in teachers noting that sometimes they have to do further check-ins with those students who contributed less.

By creating spaces for collaboration and communication, NS ASK was often seen to meaningfully contribute to supportive relationships between students and teachers and a stronger sense of self, community, and belonging.

#### 3.2.2 Theme 2: opportunities for, and experiencing success in, physical activity

Teachers highlighted how they perceived NS ASK lessons to provide new opportunities for, and allowed students to experience success in, PA. Central to the overall purpose of NS ASK, teachers perceived these lessons to include significantly more movement than traditional classroom learning, especially if outdoors. Teachers thought the lessons allowed students to practice, demonstrate strengths, and learn new skills physically. Increased movement was said to occur through various movement types, most commonly locomotor activities (i.e., running, walking), and the breaking up of sedentary time. Teachers also reported being more intentional about incorporating movement throughout the school day, by encouraging students to get up from their desks more often. This additional daily movement was noted as an extra bonus for students who do not have regular or any opportunities to engage in organized team sports or extracurricular activities:

If they're not having that opportunity to do organized sports…if it's a financial or time or whatever thing, it's good for them…to learn how to take turns, to play on a team…they get some of that in school, but it's an academic kind of structure. But with ASK, it's a little bit more sports-related PA. (T5, ES)

Additionally, teachers perceived NS ASK to offer opportunities for students to explore PA and different movement skills, such as jumping, throwing, lifting, or using sporting equipment, in a more accessible and supportive environment:

So, I find there's a huge amount of collaboration skills that come through ASK and all of our kids don't play team sports or anything now in my class, so it's their only time they're actually part of a team…or even interacting with sports equipment. (T1, ES)

Over time, teachers perceived improvements in students' physical abilities, endurance, and willingness to engage in movement: *“As the year has gone on, they're getting better at moving…getting quicker at it… I think just because they don't want to be the losing team.” (T4, MS)* A finding more typically mentioned by middle school teachers was that they experienced some students who did not want to go outside or move to learn. This was sometimes resolved by staying indoors for NS ASK or setting expectations for how to engage in the lessons early on.

Through this added opportunity for PA and practicing physical skills, many students demonstrated strengths that might not otherwise be recognized in traditional classroom lessons. This shift in perception fostered confidence, motivation, and personal growth, particularly for students who lack regular PA experience.

#### 3.2.3 Theme 3: the joy of learning with movement

This theme encompasses how teachers emphasized that movement and specifically outdoor time during NS ASK lessons brought perceived joy, happiness, emotional regulation, and improved mental wellbeing, though some students initially experience challenges adapting to this approach. Teachers emphasized that movement and outdoor lessons led to happiness. For example, one teacher shared: “*one kid even said..‘It's like she's tricking us to learn.’ And you're like, well, no, we are learning. Like they like learning that way. They're having fun.” (T1, ES)* Another added:

They come in and they seem happier…they're happy that they got outside and they look forward to it. They love it..They ask when they can go back out and do another one, so it's obviously something that they really enjoy doing. (T2, ES)

Teachers also noticed that learning while moving outdoors appeared to improve students' emotional regulation. This was especially common with exposure to the fresh air for those who are socially or emotionally heightened. One teacher even expressed that they believed the extra PA through NS ASK lessons could help reduce mental health challenges: “*the exercise, you can't beat it. Exercise battles anxiety, depression, which is running rampant in these junior high kids these days.” (T3, MS)* Overall, the combination of movement and being outdoors sparked perceived improvements in students' mood and emotional and mental wellbeing. This positive experience often was noted to translate into excitement about learning.

Despite these mostly positive observations, teachers described a few instances of negative reactions. For some, it was the initial reluctance to participate due to the novelty of this type of learning: “*There were a couple kids who refused the first couple times, especially in the [older grades], but then… the more we did it…the more it just became part of the norm.” (T7, ES)* For others, challenges arose when the lessons were framed as competitive; one middle school teacher described that some students felt anxious during faster-paced activities. One student often became frustrated or upset if her team lost or if others didn't perform well, though the teacher noted she was gradually learning to manage these reactions as she matured. While the perceived impacts were not universally positive, noted emotional benefits were more likely when teachers minimized the competitive nature, set clear expectations, and showed patience as students adjusted to the new approach.

#### 3.2.4 Theme 4: sharpening engagement and focus

NS ASK lessons were largely perceived to enhance students' engagement and focus, both during and after the lessons, supporting attention, motivation, and readiness to learn, while others also expressed some unfocused behaviour. Many teachers described that students are not only more attentive during lessons but also more eager to come to school or to specific classes:

“They're always looking forward to the next [lesson]… When you've got a group of teenagers who have had [behavioural and attendance issues] who are coming in and looking forward to the next day—that's a win. Their attendance has been perfect…they want to be outside.” (T8, MS)

While all students can benefit from increased focus, teachers particularly noted NS ASK's positive influence for students who typically have difficulty maintaining focus or attention in a traditional classroom environment. Students are engaged during the lesson because they like it and they have to keep up, as one teacher (T3, MS) describes switching up the lesson or instructions so fast, requiring the students to pay close attention. Focus was also perceived to improve when students were able to move around while they were learning, where a teacher explained that:

One [student] comes to mind, where she says, ‘I just love this because I can focus, I can move around…when I'm feeling fidgety', that it's ok outside [doing their lesson]. (T8, MS)

This was especially meaningful for students who typically struggle in a traditional classroom lesson:

Students who generally are dysregulated, [or who] come with a label or a stigma against them…it gives them an opportunity to be seen in a different light… and then they find value in themselves. (T10, MS)

Teachers shared stories from parents on how they perceived NS ASK to help students learn better through hands-on, active, and outdoor components. One teacher (T5, ES) recalled a parent telling them that their son loved ASK lessons because they helped him learn better.

Teachers also described a perceived cognitive refresh, or students being more awake and ready for the day after a lesson, such that one teacher stated:

I do think it helps them cognitively when I do the active [NS] ASK activities in the morning…I find when they come in, they're just half asleep still. So, it wakes them up, it peps them up. (T4, MS)

Many reported fewer interruptions to learning during the lessons and throughout the rest of the day, which they attributed to the extra social interaction they got during the lessons. The most mentioned perceived benefit after an NS ASK lesson was that students are typically focused, calm, and ready to learn. A teacher highlighted: “*if I do these kinds of lessons…students are more able to concentrate when they're in class. They're more focused when they get back in. They're ready to learn.” (T1, ES)* On the other hand, one teacher did express the challenge of keeping students on task during the lessons, as a new environment can sometimes cause distractions and some mentioned having students that were excited after a lesson and needed more time to settle back into learning.

Overall, teachers perceived NS ASK lessons as a reliable way to boost students' engagement and focus for better learning when they practice delivering lessons, provide clear expectations, and are willing to experiment. These findings came through especially for students who face challenges or stigmas in school, with positive effects that were perceived to extend well beyond the lesson itself.

#### 3.2.5 Theme 5: pedagogical approaches as catalysts for wellbeing

Alongside describing their perceived student experiences, teachers reflected on how those aspects of wellbeing emerged. They described pedagogical approaches, such as finding opportunities to integrate more movement throughout the day, creating more peer learning opportunities, being more adaptable and flexible in a low-pressure environment, and supporting outdoor and non-competitive lessons as much as possible, as potential pathways to wellbeing.

Teachers noted the benefits of peer learning during lessons, where students learned from and with one another rather than relying solely on teacher instruction. As one teacher put it: “*they learn better when they're up and moving around and learning from each other instead of always listening to me trying to teach them a concept or review a concept with them.” (T2, ES)* Another teacher (T6, ES) described how students learned by observing or following peers in a low-pressure context, where they might not have known the answer themselves.

Another important insight was that NS ASK lessons were said to be suitable for all students through their adaptable and flexible nature. For instance, they challenged high-achieving students to think differently and rely on peers, promoting problem-solving and flexibility, as one teacher noted:

Your top end students…a [NS] ASK activity isn't going to hurt them. It actually might change their thinking and make them do something a little bit differently because now they have to rely on a group member. So, you're also challenging them in a different way…. (T9, MS)

Teachers also expressed that NS ASK lessons are intentionally adaptable to meet diverse student needs, abilities, and preferences. They described the approach as flexible, allowing students to engage with content in ways that suit their individual strengths, learning styles, and interests. For instance:

If there's children who struggle with pen to paper and they're in a group, I can say, OK, I need to watch this kiddo today and see, ask questions, and then I do use it for them to show what they know… There is a child that I based his entire report card off situations like this because that's where he thrives. (T10, MS)

Lessons were also seen as low-pressure spaces where students could revisit and practice skills without the stress or formality of traditional instruction, particularly when offered as non-competitive. One teacher expressed: “*I like how easy it is to assist students using it and how it reinforces learning in a positive, happy way for them.” (T1, ES)* These flexible and inclusive characteristics of NS ASK, including movement and outdoor time, were seen by teachers as key contributors to supporting students' wellbeing in many domains.

#### 3.2.6 Theme 6: ‘running' out of resources

This theme describes the resource and support challenges that teachers and their schools faced in delivering NS ASK lessons. Teachers described spatial constraints due to small indoor spaces, overcrowded classrooms, limited access to alternate indoor or outdoor spaces, and weather conditions preventing outdoor use. They also expressed a perceived lack of equipment, time for preparing lessons, and adequate support for students with disabilities. One teacher succinctly explained the issue of finding space for lessons:

[We have] a small older school. The classrooms are an OK size, nothing for an [NS] ASK program though, which [is] a problem in bad weather… because I can't do it in my classroom. It takes a major upsetting of moving chairs and desks, and I can only do it with certain classes because of gym availability. (T3, MS)

While space was a key issue, many teachers also spoke about a lack of durable or sufficient NS ASK equipment for the whole school. Although NS ASK encourages simple lessons that often require little to no equipment, shared items like dice, numbered or lettered bean bags, cones, or hula hoops were in high demand, complicating last minute lesson planning. As one teacher said:

If I'm going to use [NS ASK materials] … I usually go first thing in the morning or right before recess and claim it or even the day before if I know I'm going to use it multiple times, I get it just before someone else uses it. (T2, ES)

For those who had equipment, some discussed the ongoing maintenance involved, often using their own money to replace them. Additionally, ensuring students had appropriate outdoor clothing was a frequent challenge, as one teacher noted:

Sometimes students arrive without the right boots or jackets, but I was still going out because I told them that would happen. So pretty soon after…they're cold, they remember the jackets the next day. (T1, ES)

Time was another challenge. Teachers described the demands of preparing a NS ASK lesson, adapting the curriculum to be active, managing competing demands, and gathering or creating materials. One teacher (T3, MS) described how competing demands take away from their planning time, especially when they have to cover for other colleagues during their preparatory periods. Although teachers valued NS ASK's online lesson database as a way to support planning, some found it unintuitive or not user-friendly, and suggested adding visuals (e.g., videos) and editable forms for easier adaptation.

Lastly, inclusivity was also a concern, especially for students with disabilities. One teacher reflected:

I have a student coming into my class in the next few years who is in a wheelchair.. while the [main path] is accessible to a wheelchair, anything off the path [where I usually set up] is not accessible. (T8, MS)

More work is needed to fully support all students in their ability to take part in PAL. Each of these challenges highlights the need for adequate space, equipment, time, and accessible planning tools to support teachers in delivering NS ASK lessons. While teachers found ways to adapt and persevere, greater resources, infrastructure, and support would enable more consistent and inclusive NS ASK delivery.

#### 3.2.7 Theme 7: navigating the status quo

This final theme captures how teachers navigated the realities of conducting NS ASK lessons within existing school structures, systems, and cultures (i.e., collective attitudes, routines, and beliefs within the school). For many, NS ASK required a mindset shift, for themselves, their students, and other teachers and staff, away from familiar, structured, and classroom-based forms of learning toward more flexible, experiential approaches. Several teachers noted that they were the only ones implementing NS ASK in their schools, while others faced skepticism from colleagues who viewed NS ASK as unstructured or non-essential. As one teacher reflected, “*some barriers might be other teachers…they see a younger teacher doing something not in the classroom that doesn't look like learning… just getting some people to get over that can be a barrier.*” *(T9, MS)* When teachers do not feel supported in their endeavours, it is difficult to continue.

Teachers also described balancing NS ASK with curriculum pressures and traditional assessment expectations. As one teacher explained, “*It's harder to assess them academically…you can make it part of their mark a little bit.. like it's fun and can be part of their grade and their participation.” (T4, MS)* This required teachers to think differently about what assessment could look like during movement-based learning and to identify when and how NS ASK best fit within their instructional days.

Additional factors that are not as common in status quo, traditional teaching, such as ensuring student safety and teachers' confidence or comfort with outdoor learning, further shaped how NS ASK was taken up in practice. Despite these challenges, many persisted because they saw clear benefits for students. Still, embedding NS ASK more fully will require not only individual teacher adaptation but broader cultural and structural shifts across school systems.

## 4 Discussion

This study used semi-structured interviews to inductively explore elementary and middle school teachers' perceptions of the NS ASK project, focusing on student wellbeing and the challenges of implementing this PAL program. Seven themes were developed: four related to the perceived physical, cognitive, social, and emotional/mental wellbeing benefits; one surrounding the pedagogical approaches perceived to support these benefits; and two highlighted ongoing challenges of implementation. Our findings align with the three basic needs of the SDT (33) and with nature relatedness as a proposed fourth need (34). This expanded SDT provided a useful lens for interpreting our findings and situating them within the broader PAL literature that has integrated the SDT (25, 43, 44) as a way to describe students' motivation, engagement, and wellbeing (45). These concepts help contextualize why teachers may have observed the impacts of NS ASK lessons on students' wellbeing. Figure 1 visually connects the expanded SDT needs to the identified pedagogical approaches and the four domains of wellbeing outcomes, interpreted using the UNICEF and WHO/UNESCO wellbeing domains (28, 32) after theme development. The arrows represent that satisfying these needs through the noted NS ASK approaches may contribute to one or more of the perceived benefits.

**Figure 1:**
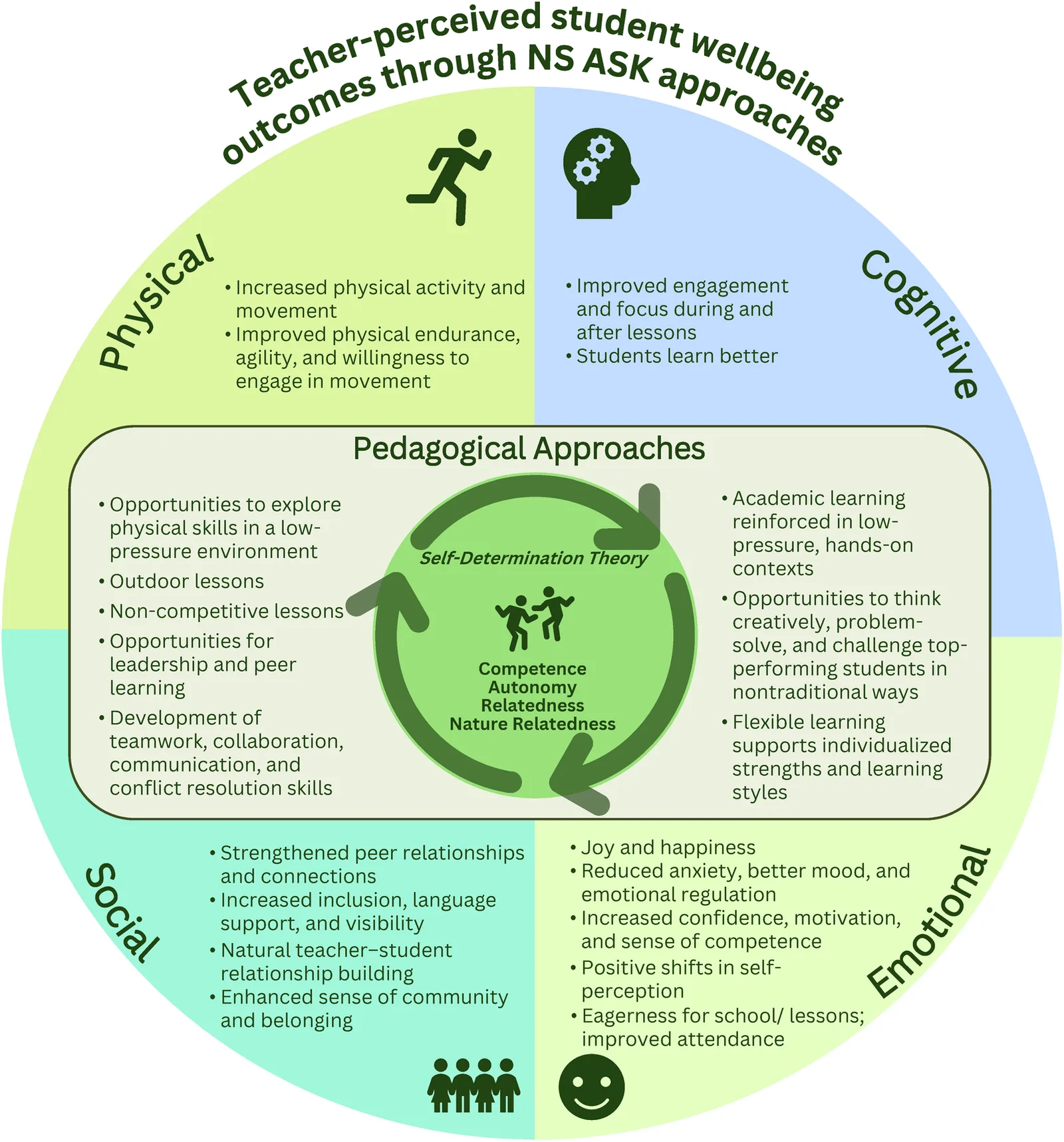
Visual depicting teachers' perceptions of the Nova Scotia Active Smarter Kids Project's contribution to students' wellbeing (physical, cognitive, social, emotional) and pedagogical approaches for supporting these outcomes (interpreted ùsing and connected to Self-Determination Theory).

### 4.1 Physical wellbeing: inclusive, low-barrier, and outdoor opportunities for movement and competence-building

NS ASK lessons were perceived to impact physical wellbeing by offering inclusive, low-barrier opportunities for PA and breaking up sedentary time throughout the school day. Unlike PE, which can feel evaluative and skill-based, teachers described NS ASK as a space where students could participate and experience success regardless of prior athletic ability (46), connecting to feelings of competence and autonomy. Such inclusive, competence-building opportunities align with findings from other PAL studies that found that low-academic achieving students had their physical competence recognized and valued (47, 48), highlighting how movement itself can create contexts for success in PAL.

Teachers also shared that NS ASK seemed valuable for students who might not have access to organized sports or extracurricular activities, offering them a chance to engage in PA during the school day. Importantly, in line with another PAL study (49), teachers in our study noted that some students developed new physical skills or improved fitness over time through regular participation. While ASK in Norway demonstrated improvements in fitness with mixed effects on health markers (50), our findings connect to NS ASK's potential contribution to students' physical literacy by creating inclusive competence, confidence, and motivation-building opportunities in everyday classroom contexts (51).

NS ASK lessons often take place outdoors, which can also support aspects of physical wellbeing. Although outdoor spaces allow for a range of behaviours, recent evidence continues to show that, on average, children accumulate more PA outdoors compared to indoors (52–54). Exposure to nature through these lessons may also help strengthen students' nature relatedness, which has been linked in some cross-sectional studies to greater motivation for and participation in PA (55, 56). However, these associations are very dependent on children's experience with nature and the outdoors. Nevertheless, this additional reported opportunity for outdoor PA during the school day may further help students work toward the Canadian 24-hour movement behaviour recommendations (57), and, in Nova Scotia, the *Physical Activity Framework* guidelines (26).

### 4.2 Cognitive wellbeing: enhancing focus, engagement, and cognitive growth through active, engaging, and inclusive learning environments

Teachers perceived NS ASK to create opportunities for cognitive wellbeing, such that students were noted to succeed in ways not typically recognized in traditional lessons and to increase their engagement and focus during learning, supported by peer, embodied, and outdoor learning. Students who struggled with academic tasks, especially those with diagnosed ADHD or other learning disabilities, felt success and inclusion through active, hands-on participation, while physically skilled students often supported peers, broadening how success was defined and celebrated. The perception that students could experience competence in what they were doing, and autonomy in how they contributed, aligns with key motivational needs (33).

The philosophies associated with NS ASK, such as avoiding competition and enabling participation in PA in ways that suit them, all align with UDL, inclusive pedagogies, and strength-based approaches to teaching and learning. UDL and inclusive pedagogies aim to make learning accessible to all learners rather than creating accommodations for some (27). By including varied forms of participation and assessment, NS ASK lessons may help more students see themselves as capable contributors in the classroom, leading to greater engagement and confidence, as well as agency and resilience. This perception is consistent with findings from the ASK intervention in Norway, which showed that while no overall effect on academic performance was found, students in the lowest tertile at baseline demonstrated significant improvements in numeracy and executive functions (50, 58). These findings also suggest that PAL may particularly benefit those most in need of adapted education, even if effects are less consistent for higher-performing students.

A particularly important aspect mentioned by teachers was the use of small groups, where students could support one another's learning or take on roles aligned with their strengths. Along with the perceived social benefits, for students who typically found classroom tasks challenging, being part of a group meant they could meaningfully contribute, potentially satisfying their need for relatedness, and experience success. The appeal of group work in this context is echoed in other research; for example, Dyrstad et al. (49) reported that PAL supported lower-achieving students by creating collaborative environments where peers could help each other master academic content. Additionally, the material could be tailored to specific group needs and levels of achievement, which was also noted by Mandelid et al. (59), allowing everyone to feel mastery and competence.

Teachers also described how NS ASK often helped sustain students' focus both during and after the lessons, which they perceived to be due to enhanced engagement with content, consistent with previous research on PAL (59). Engagement itself is a critical predictor of learning outcomes, linking to better achievement, grades, and school completion rates (60), and can translate into increased time-on-task, which is a marker of behavioural engagement, as has been observed in other PAL intervention studies (61, 62). As school engagement tends to decline with age (63), the fact that the current study demonstrated perceived engagement in both older elementary and middle school ages is promising for the use of NS ASK, and PAL generally, beyond just elementary ages.

Movement and enjoyment are vital components of PAL, supporting engagement, focus, and embodied, experiential learning (64–66). Teachers highlighted the role of enjoyment in learning, with one noting that a student felt “tricked into learning” because the activity was so engaging. Enjoyable, active experiences can foster optimal cognitive conditions, aligning attention, motivation, and emotional states to enhance learning (67). Other PAL studies further illustrate the impact of movement and enjoyment on focus, where teachers noted that even students who had behavioural challenges were able to focus better after PAL (49) and that PAL kept students motivated, with higher concentration and focus during and after the lessons (68).

Teachers highlighted that being outdoors during ASK may have contributed to greater attention and focus, as well as emotional wellbeing. Humans' inherent connection to nature, known as the biophilia hypothesis (69), or nature relatedness, may, in part, explain why students prefer outdoor lessons, thereby increasing focus. However, as some students were reported to experience distractions and off-task behaviours outdoors, more research is needed on how outdoor PAL experiences on school grounds impact the focus and other related outcomes of students with different backgrounds and experiences.

### 4.3 Social wellbeing: building inclusion, connection, and belonging through collaboration

Teachers discussed many components of social wellbeing, where NS ASK was perceived to promote social inclusion, teamwork, communication, leadership, and relationships with peers and teachers. These components are key social skills and can be linked to the autonomy and relatedness components of the SDT (33). The highly social, collaborative, and supportive format of NS ASK lessons was found to be the source of connection and belonging that is sometimes missing in regular classroom environments. Even simply the addition of PA into the lessons may have supported the perceived social benefits, as engaging in movement together can aid in the development of friendships, peer relationships, and social identities (70, 71).

The idea of creating a sense of belonging came up in relation to students who struggle socially, such as those who experience learning disabilities or language barriers. Another study similarly highlighted the experience of students with immigrant backgrounds participating in education outside the classroom, noting how these sessions offered low-barrier participation, essential for feelings of autonomy and safety, where the hands-on activities allowed students who struggled with the language to be able to experience some success and feelings of competence and be connected socially with their classmates (48). This sense of belonging, visibility, and peer acceptance is crucial in academic and overall wellbeing for students now and into adulthood (72, 73).

Other PAL studies have also found that students are able to foster social connections and belonging (22, 30, 46). Interestingly, while the Norwegian ASK cluster-randomized controlled trial did not detect significant effects on students' health-related quality of life domains, such as social support and peers or school environment (74), teachers in the intervention also spoke to how ASK strengthened students' cohesion and belonging, especially for those students with low academic achievement (30). This contrast may suggest that large-scale trials using standardized Health-Related Quality of Life instruments may not capture the nuanced, relational benefits of PAL. The current qualitative study highlights teachers' observations and the everyday ways PAL lessons can foster positive peer dynamics and social integration.

Aside from just peer relationships in the class, teachers also mentioned the perceived benefit of creating stronger relationships between them and their students through NS ASK lessons. Student-teacher relationships are an important avenue for supporting feelings of belonging and relatedness in school and can be difficult to accomplish when teachers feel the pressure of covering all their required materials and fulfilling many responsibilities (75). The more playful side of learning can be fostered with NS ASK, where the original ASK study and another PAL study in Norway also highlighted that PAL provided an opportunity to get to know students in a different way and see new sides of them during these lessons (30, 59). Another study that observed the implementation of PAL found that when teachers participated in the lessons, students were more likely to be active, and for longer and at higher intensities (76). Thus, teachers getting their class involved in NS ASK and being active participants themselves could be related to both social and physical benefits.

### 4.4 Emotional wellbeing: enjoyment, regulation, and motivation through supportive environments for learning

Joy, happiness, emotional regulation, mental wellbeing, as well as competence, confidence, and motivation were highlighted by teachers as perceived emotional wellbeing outcomes supported during NS ASK. Generally, students' enjoyment with PAL has been well established in other studies, where adding movement into lessons was perceived positively (17, 46, 47, 49, 68).

Teachers frequently highlighted the connection between students' enjoyment of the lessons and being outdoors. Beyond simply being outside, outdoor learning environments can introduce novelty and perceived variety into the school day (22, 49). Perceived variety complements the basic psychological needs described in SDT and contributes to greater enjoyment and engagement in PA and learning (77, 78). In the context of NS ASK, the shift from static classroom learning toward dynamic, movement-based lessons, outdoors, but also in other indoor areas of the school, such as the cafeteria, hallways, and gym, may be an important motivational factor, keeping students interested and excited to participate.

Teachers' observations also point to the direct influence of outdoor and nature contact on students' emotional wellbeing. Stress Reduction Theory suggests that natural settings can lower stress and promote emotional regulation (79), which may partly explain teachers' observations of improved mood, school attendance, and reduced anxiety during and after NS ASK lessons. Thus, outdoor, movement-rich lessons might be particularly supportive for students' emotional wellbeing, engagement, and readiness to learn.

Positive emotional outcomes sometimes varied depending on lesson context. Other studies similarly show that factors such as time of day, students' familiarity with this type of learning, lesson location, instructional complexity, and the type of movement involved can impact emotional engagement (21, 22). In our findings, a main source of emotional disengagement was the inclusion of competitive elements. While competition motivated some students, for others, it appeared to undermine feelings of competence or autonomy, creating anxiety or reluctance to participate. This aligns with previous research indicating potential downsides of competition in PAL. Jørring et al. (47) found that competitions and relay races could foster extrinsic motivation but also create exclusion, particularly among students who struggled academically or were distracted by the competitive atmosphere. Even high-achieving students reported difficulty focusing due to pressure from teammates (47). Similarly, Aadland et al. (70) noted that while group activities often foster cooperation, an overemphasis on competition may reduce relatedness and wellbeing for some children. These findings reinforce broader evidence that supportive, low-pressure learning environments are important for students' emotional wellbeing and sense of autonomy, competence, and relatedness (80).

### 4.5 Challenges of implementing Nova Scotia Active Smarter Kids

Teachers' perceived challenges when implementing NS ASK were largely aligned with those identified in other similar studies from around the world, such as lack of time, space, resources, administrative support, and school culture (59, 68, 81, 82). As this study only included teachers who currently deliver NS ASK, some common barriers, such as perceived lack of confidence or self-efficacy, did not emerge, as these teachers had already overcome them and were motivated by their educational values or perceived benefits for students. Notably, none of the teachers reported having training in PAL or outdoor learning during their teacher education, and only half reported expertise in PA, PE, or health. When teachers spoke about challenges related to time and space, these were not typically about a lack of personal willingness to adapt; teachers described creative strategies such as borrowing equipment early, rearranging classrooms, and collaborating with colleagues. These challenges reflected broader systemic constraints, including staffing shortages, overcrowded classrooms, and an increasingly packed curriculum that leaves little flexibility.

One challenge that has received less attention in prior literature was the difficulty of implementing PAL with students who have physical disabilities. While teachers described adapting lessons more easily for students with intellectual or learning disabilities, some faced, or anticipated facing, greater challenges when trying to include students with mobility, vision, or hearing impairments. For example, Quarmby et al. (82) highlighted a teacher's account of her high school-aged son, a wheelchair user, who was often excluded from PAL activities or assigned to work with other socially marginalized peers. This underscores the risk that PAL, if not carefully planned, could inadvertently isolate students with disabilities.

### 4.6 Implications for practice and policy

This study highlights the potential for NS ASK to positively influence elementary and middle school students' wellbeing by supporting essential skills and behaviours that can impact lifelong health (32). The study also noted challenges, leading to recommendations to better support NS ASK and PAL in similar educational contexts, including the need for educational policymakers, school space designers, educators, school administrators, and other decision-makers to act collectively for improved lesson planning resources; administrative support that values and prioritizes PAL; investing in infrastructure for flexible, accessible spaces; providing durable equipment and targeted professional development, particularly around inclusive practice with students with disabilities; developing policies that prioritize and protect time for PAL; and ensuring sustainable funding from school regions and government education departments (12).

Recent work has shown that implementing PAL is not simply a teaching strategy or a competence to be taught in isolation. Instead, it is deeply connected to teachers' broader values, educational philosophies, and professional judgments (59, 83). Quality and sustainability depend on supporting teachers in exploring why and how PAL aligns with their educational goals. Teachers in this study described how school-wide norms, culture, and structures shaped their ability to implement NS ASK. These insights provide a nuanced understanding of systemic influences on uptake, consistent with prior research on whole-school and systemic strategies supporting PAL (12, 84). Introducing PAL early in students' school experience helps integrate movement as a normative practice rather than an exception (22), and pre-service teacher education should explicitly incorporate PAL principles, to frame these strategies as foundational rather than optional (16, 85).

Countries such as Norway and the UK have leveraged existing curriculum policies emphasizing student social development, PA, and respect for nature to justify PAL integration (16). While teacher commitment is strong, sustainable and equitable delivery of PAL ultimately depends on multi-level, coordinated efforts to remove systemic and structural barriers. The positive perceived benefits in this study, including improved engagement, focus, readiness to learn, and socio-emotional outcomes, can inform policy for NS ASK and broader PAL initiatives, highlighting the value of embedding PAL within curriculum planning and promoting consistent, system-level implementation in Nova Scotia and beyond.

### 4.7 Strengths and limitations

Strengths of this study include the co-development of the interview guide and study logistics with a steering committee of education and health professionals familiar with NS ASK, ensuring relevance and meaningfulness for teachers and knowledge users. The study also captured rich perspectives from teachers across elementary and middle schools in different school regions, with varied years of teaching experience, school sizes, urban/rural locations, grade compositions, and lengths of involvement in NS ASK. This diversity of perspectives and contexts enriched the data and enhanced transferability of findings.

A limitation of this study is that all participants were already engaged in NS ASK, thus only providing the perspectives of relatively motivated and experienced teachers. Additionally, all identified as White European, and most were women. These participant characteristics highlight the need for future research to include teachers of different ethnicities, cultural backgrounds, and genders who might have different views surrounding teaching, movement, and outdoor time (86), as well as those less familiar with, less supportive of, or not implementing PAL, to better understand barriers to adoption and for greater transferability of findings. However, consistent with our RTA approach, interpretation and analysis aimed to represent the range of ideas and experiences of those engaged in this research, and since this analysis was contextually situated, the findings are representative of this sample and are not meant to be universally generalizable, but can provide insights for similar contexts (38). Lastly, focusing solely on teachers also limited insight into student experiences and perceptions, though follow-up research has been completed to capture student perspectives with NS ASK.

## 5 Conclusion

NS ASK lessons were perceived by elementary and middle school teachers across Nova Scotia, Canada, to have meaningful physical, cognitive, social, and emotional wellbeing benefits for students. These findings are connected to the perceived pedagogical approaches of NS ASK such as the flexible, low-pressure, non-competitive, often outdoors, and team-based nature of the lessons that satisfy the basic psychological needs of the SDT (33) and nature relatedness (34). The perceived benefits of NS ASK were also found to align with international child and youth wellbeing frameworks such as UNICEF (32) and WHO and UNESCO's HPS standards (28), and local educational frameworks for movement, outdoor time, and overall student wellbeing.

These teachers, who often act as champions of PAL, have demonstrated creativity and dedication in embedding PAL into their teaching practice. However, they also continue to face challenges in starting and sustaining implementation. Realizing the full potential of NS ASK, and other PAL approaches in similar education contexts, requires addressing structural challenges and fostering shifts in educational culture through whole-school approaches, pre-service teacher education, protected time for teacher preparation of lessons, user-friendly resources for teachers, and sustained funding models. If this can be achieved, more students will be able to reap the benefits of NS ASK and PAL.

## Data Availability

The underlying data cannot be shared due to ethical restrictions that protect the privacy of the participants. The data will be shared on reasonable request to the corresponding author and upon signing of a data transfer agreement. Requests to access the datasets should be directed to michelle.stone@dal.ca.
