# Supplementary material for: Teachers' perspectives on physically active learning and student wellbeing in the Nova Scotia Active Smarter Kids Project
Source: Front Sports Act Living. 2026 Oct 14;8:1914074. doi: 10.3389/fspor.2026.1914074 (PMC13713629; doi:10.3389/fspor.2026.1914074)
Supplement: Supplementary file 1 [file Supplementaryfile1.docx]

Supplementary Material

**Supplementary file #1.** Teacher demographic questionnaire and interview guide

*Demographic questionnaire*

a. What is your age category (<35, 36-50, 50-65, >65 years)
b. How would you describe your gender?
a. How would you describe your cultural background/Ethnicity?
a. Do you identify as someone who lives with a disability?
a. How long have you been teaching?
   1. At this school/this grade?
b. Do you have any teaching experience or other qualifications in Physical Education/Health/Physical Activity? If so, what?
c. How was physically active learning or outdoor learning included in your teacher training? Were you familiar with these approaches?

*Section A: First, I’m interested to know a little bit about your school and your class…*

1. What grade(s) do you teach?
2. How many students are in your class?
3. How big is your classroom? Is there room to move?
4. Are there other spaces indoors or outdoors where you can teach?

*Section B: The next section is about your experience with Nova Scotia Active Smarter Kids (NS ASK), like when you were trained, why you decided to implement it in your classroom, and examples of lessons you use…*

5. When and where were you trained in ASK (by a trained NS ASK facilitator)?
   i. How many training sessions have you participated in?
6. How long have you used ASK lessons in your classroom? Did you do anything similar before being trained in ASK?
7. What was your main motivation for implementing ASK in your classroom?
8. Tell me about some ASK lessons that you do, specifically surrounding how often you do them, where they take place, how long they are, what subjects you teach, and the general purpose of the lesson…

**Prompts:**

i. How often do you conduct ASK lessons? Daily, weekly, monthly…?
ii. How long are the lessons usually?
iii. Where do you conduct ASK lessons; for example, in your classroom, foyer, or outside?
     1. Do you have an outdoor classroom, and is it used for ASK lessons?
        a. Does the outdoor classroom facilitate or hinder ASK lessons?
iv. What subjects do you typically include?
v. What is the general purpose of the lessons?
   1. Formative assessments? To re-engage students? Learning new material?

*Section C: Next, we will get into how the students get along with ASK lessons, specifically surrounding their social, emotional/mental, and cognitive outcomes*

9. When thinking about students both during and beyond ASK lessons (back in classroom), what have you noticed about ASK lessons and how they might contribute to or take away from these factors?

**Prompts:**

i. During and beyond ASK lessons.
ii. Cognitive?
    1. Focus
    2. Engagement
    3. Learning outcomes
iii. Social?
     1. Friendship
     2. Belonging
     3. Leadership
     4. Teamwork
iv. Emotional/Mental?
    1. Anxiety
    2. General happiness
    3. Wanting to come to school/your class
    4. Behavioural issues
v. Do these differ based on the location of ASK lesson? Indoor/outdoor? Classroom vs. anywhere else?
10. What have you noticed about the types of students who benefit most/least from ASK lessons? Can you provide an example?

**Prompts:**

i. Is there a student who struggles with sitting in class?
ii. A student who usually does not take part in other PA opportunities?
iii. A student who struggles with learning concepts in the classroom?
iv. A student who thrives in the traditional classroom?
v. A student with disabilities?

*Section D: Great, now we will move on to the physical health side of things…*

11. In terms of students’ physical activity, what have you noticed in relation to ASK lessons? Like the types of movements, intensities, and how it compares to traditional indoor classes?

**Prompts:**

i. You mentioned the lessons are typically [# minutes long], how much of that time are students usually moving?
ii. What is the movement intensity during an ASK lesson? Light, moderate, vigorous?
iii. What types of movement are usually carried out? Walking, running, jumping, throwing…?
iv. How does their movement compare to when you conduct a typical lesson in the classroom?
12. Great, you’ve talked about movement during ASK, but do ASK lessons have any effect on students’ movement outside of ASK lessons, like when back in the classroom or during recess?

**Prompts:**

i. Are you adding in other movement throughout the day?
ii. Are students moving more at other times like at recess?
13. How is sitting time affected by ASK lessons?

**Prompts:**

i. During the ASK lesson? Is sedentary time being broken up more?
ii. Beyond the lesson?
    1. How are children’s initiatives/behaviours affected?
    2. How are your teaching practices affected?
14. Have you noticed any changes in students’ confidence or motivation to engage in physical activity through ASK lessons? What are some examples of what you’ve noticed?

*Section E: The next section will touch on challenges and barriers associated with ASK…*

15. Tell me about any challenges or barriers you've experienced when initially adopting/implementing ASK

**Prompts:**

i. School/administration support, space, time…
16. What about any current challenges or barriers as you are conducting ASK lessons?

**Prompts:**

i. Preparation for the lessons, school/administration support, finding space, accessibility concerns…
17. How could you overcome these challenges/barriers? What might help?

**Prompts:**

i. More training/support from NS ASK, equipment, lesson plans, evidence on benefits for regional/school support, resources for accessibility, information for families…

*Section F: Some last questions if time allows/they have not been brought up yet…*

18. Generally, how has conducting ASK lessons changed your teaching practices?

**Prompts:**

i. How has your classroom management changed? During ASK lessons? While in the classroom?
ii. How has your lesson planning changed? Time spent?
19. Is your school community (principal, other teachers, school advisory council, educational assistants, custodians, parents) supportive of ASK?
20. How has conducting ASK lessons impacted your health and wellbeing, both at school and at home?
21. Lastly, is there anything else you would like to share about ASK lessons and your experience or student health and wellbeing outcomes?
