# Supplementary material for: Teachers' perspectives on physically active learning and student wellbeing in the Nova Scotia Active Smarter Kids Project
Source: Front Sports Act Living. 2026 Oct 14;8:1914074. doi: 10.3389/fspor.2026.1914074 (PMC13713629; doi:10.3389/fspor.2026.1914074)
Supplement: Supplementary file 3 [file Supplementaryfile3.docx]

**Supplementary file #3: Theme summaries and code table with representative quotes and connection to wellbeing domains**

| **Code Name** | **Description** | **Example quote** | **Wellbeing domain** |
| --- | --- | --- | --- |
| Theme 1: Fostering belonging and relationship building | | | |
| This theme explores the way in which NS ASK lessons provide opportunities for students to socialize in their often-collaborative formats, forming new relationships and connections with other students and their teacher, and fostering feelings of belonging and leadership, whereas some teachers also perceived some negative social impacts. | | | |
| New relationships | New relationships are fostered, often through intentional grouping and partnerships, that encourage students to interact with peers outside their usual circles. | “They're talking to people that maybe they wouldn't always talk to, and now they're starting, they start talking about the math and all of a sudden a couple minutes later, after the activity is done, now they're talking about what they're doing on the weekend and then starting to form those relationships.” T9, MS | Social |
| Connection with peers | Connection through shared experiences, socializing, collaboration, and empathy. | “For this particular lesson, we brought them down and then we put them in partners, and they had to build a word to describe the other person, and we did that a couple times with the classes which was really nice to build that sense of community.” T7, ES | Social |
| Student teacher connection | ASK creates natural opportunities for teachers to connect meaningfully with their students through shared participation and informal interactions. | “It's really good for relationship building at the beginning of the year and then maintaining those relationships throughout the year…If you're going to get any student to listen to you or respect you…you should know them in school and a little bit outside of school and they should know who you are as a person too. And in…the structure of an ASK lesson, it's good. You can…have those side conversations which are really nice to build that relationship.” T5, ES | Social |
| Belonging | Students, particularly those who might often be on the margins (due to learning needs, social differences, or language barriers), feel more engaged, visible, and part of the group. | “There's a lot of language barriers, so when I make up a game…I'll put the question just in their language. So, they'll feel more involved instead of just an English question that they wouldn't know the answer to, so everybody has to rally around them because they're the only one that knows how to speak that language and answer the question.” T3, MS | Social |
| Leadership | Teachers encouraging leadership or naturally emerging leadership among students, such as guiding peers and taking initiative. | “Yeah, they feel good about themselves because they're kind of leading the jock kids, like they've realized they've really helped their group because they understood the assignment.” T3, MS | Social |
| Learning about yourself | Through collaborative ASK lessons, students learn about their abilities, strengths, limitations, and who they work well with. | “Most importantly, it’s about them being able to figure out their strengths but also know what their limits are and who they can work with…It’s not about [the specific lesson] …it is ‘what have I learned about myself? ’What do I bring to the team?” T8, MS | Social |
| Students letting others do their work | Some accountability challenges, where stronger students tend to take the lead while others disengage or rely on them to complete the work. | "Again, you know there needs to be that whole accountability thing even within the group that one person doesn't just take over and do everything right…Your goal is that they will work together, help each other and make it so that everybody in the group is more important than just one person getting everything right." T6, ES | Social (-ve) |
| Theme 2: Opportunities for, and experiencing success in, physical activity | | | |
| This theme highlights how teachers perceived NS ASK lessons to provide new opportunities for, and allowed students to experience success in, PA. | | | |
| Movement | ASK lessons add more movement than traditional classroom learning. | “[ASK lessons are] very much a cardio based activity. Versus the traditional just kind of sit down, get up to move around in the classroom a little bit. They're not doing any of the running or the jumping or the throwing. No, they're not doing any of that in the actual classroom when we're covering the material. They might get up, move around to do a group problem here…But again, they're not doing that truly cardio based physical activity.” T9, MS | Physical |
| Different movements | ASK provides the opportunity to engage in different movement types, especially locomotor movements. | “It’s lifting, it’s dragging, it’s hauling, it’s running, it’s squatting, it’s crawling in under, it’s being able to get into crammed spaces once the shelter’s done because every team member has to be able to fit into the shelter once it’s done.” T8, MS | Physical |
| Breaking up sedentary time | Teachers are mindful of breaking up sedentary time throughout the day, even outside of ASK lessons. | “Instead of having them sitting at their desks for all three parts, [I would say] “come and join me at the meeting area...You can bring your chair, but pick up your chair and come walk. Sit. OK, now we're going to go do a pair and share walk. Okay, come back...Pick up your chair. Go back. It's time to work.” 15-20 minutes later... “stand in a circle and we're going to share our [work]”... I know this is not technically an ASK lesson, but it is the purposeful, intentional [decision] in getting some movement and interrupting sitting time.” T7, ES | Physical |
| Supplements sports and extracurricular activities | ASK provides physical activity and sport-like experiences, such as learning new movement skills, for students who may not participate in organized sports or extracurriculars due to barriers like cost, time, or interest. | “If they're not having that opportunity to do like organized sports…if it's a financial or time or whatever thing, it's good for them…to learn how to take turns, to play on a team…they get some of that in school, but it's an academic kind of structure. But with ASK, it's a little bit more sports-related physical activity.” T5, ES | Physical |
| Getting better at moving | Changes in students' physical activity ability, endurance, or willingness to engage in movement over time during ASK. | “As the year has gone on, they're getting better at moving. They're getting quicker at it. You know, I think just because they don't want to be the losing team.” T4, MS | Physical |
| Students not wanting to move or go outdoors | Some students did not want to move or go outdoors to learn. | “There are some kids that will just not participate. They're not active, they just want to be left alone. Luckily, I can count those kids on one hand that have refused totally to participate in it.” T3, MS | Physical (-ve) |
| Theme 3: The joy of learning with movement | | | |
| This theme encompasses how teachers emphasized that movement and specifically outdoor time during NS ASK lessons brought perceived joy, happiness, emotional regulation, and improved mental wellbeing, though some students initially experience challenges adapting to this approach. | | | |
| Happiness | Happiness and positive mood during and after ASK lessons. | “Emotionally, yeah, I feel like typically they come in and they seem happier and they're happy that they got outside and they love it. So they look forward to it. They love it. They want to go outside. They ask when they can go back out and do another one, so it's obviously something that they really enjoy doing.” T2, ES | Emotional |
| Regulation | Improved behavioural/emotional regulation when learning outdoors or through physical activity. | “Also, fresh air is really, really good. I've had students talk about that. It kind of helps students that are heightened if they're socially or emotionally a little unstable. If they are just a little heightened and they need to come down, same with like running around and being active.” T5, ES | Emotional |
| Battling depression and anxiety | Adding in extra physical activity through ASK lessons can aid in reducing depression and anxiety. | “And again, the exercise, you can't beat it. Exercise battles anxiety, depression, which is running rampant in these junior high kids these days.” T3, MS | Emotional |
| Enjoying learning | Student enjoyment, excitement, and engagement with learning during ASK lessons, and it not feeling like learning. | “One kid even said to me once: “It's like she's tricking us to learn.” And you're like, well, no, we are learning. They like learning that way. They're having fun.” T1, ES | Emotional |
| Enjoying at all ages | Older students are enjoying and asking to participate in ASK lessons. | “And it's funny…I'm going outside with the equipment and [sometimes there are other older kids that see me and say:] “ohh, we want to come out and do those… Why doesn’t our teacher do them?” So kids still love it as they get older and they still want to be doing it. And a lot of times, if I'm out there with my class doing something and there's older kids, they’ll ask if they can come and play too, and then they want to ask the math questions, and my kids think it’s hilarious. You know, junior high kids are now playing the game.” T1, ES | Emotional |
| Asking for ASK and motivated | Students are enjoying the lessons so much that they are asking for them and wanting to know when the next one is. | “Oh yeah, I get asked all the time “Are we doing an ASK lesson today?” “Are we going outside?”” T5, ES | Emotional |
| Not liking it at first | Initially, some students did not enjoy ASK lessons. | “There were a couple kids who refused the first couple times, especially in the [grade] sixes, but then…the more we did it and the more it just became part of the norm…” T7, ES | Emotional (-ve) |
| Anxiety | Some students experience anxiety in taking part, especially when the lessons are competitive in nature. | “I know when I do the [ASK lesson], sometimes the kids who can't answer things fast get…a little bit of anxiety because they're like, “I know it, I just can't answer it that quick.” So, I'm always saying [to] the kids “don't rush them. You get more points and I give points for the most correct answers, not who finishes first.” So, I'm like, you get more points for taking your time, so don't feel like you have to rush.” T4, MS | Emotional (-ve) |
| Issues with competitive nature emotionally | Some students struggle with a lesson if it is competitive. | “Yeah, I have one girl…She just doesn't ever want to be last…She has major anger issues, in a sense. And if she if she doesn't do well, she gets upset. She’s getting so much better. She's maturing as she gets older, but yeah, she would be like “[teacher name], we lost because they didn't do [this or]…know that question or whatever” T4, MS | Emotional (-ve) |
| Theme 4: Sharpening engagement and focus | | | |
| NS ASK lessons were largely perceived to enhance students’ engagement and focus, both during and after the lessons, supporting attention, motivation, and readiness to learn, while others also expressed some unfocused behaviour. | | | |
| Engaged | Students are engaged during the lesson because they like it and they have to be to keep up. | “They have to keep their eye on me and listening skills because I'm switching up so fast too.” T3, MS | Cognitive |
| Happy to come to school and class | Students are eager and willing to come to school or specific classes due to ASK lessons. | “Yeah…they’re always looking forward to the next, ‘what are we doing next day?’ ‘Are we continuing with the forest or are we doing something else?’ To me, when you’ve got a group of teenagers, who a lot of them have [behavioural difficulties] and things like that, who come in and are looking forward to the next day, that’s a win. Because their attendance has been perfect, and it’s flu season, and everybody knows that it’s flu season, but they are looking forward to being outside.” T8, MS | Emotional |
| Focus during lesson | Students are focused on the learning during ASK lessons. | “One [student] in particular comes to mind, where she comes to me and she says ‘I just love this because I can focus, I can move around, you know when I’m feeling fidgety’ that it’s ok…outside building the shelter because you know, I’m asking you to go and move and bring more sticks or do something like that.” T8, MS | Cognitive |
| Opportunity to shine | Students are able to demonstrate their strengths and engage/excel in the learning, especially those who may struggle in a traditional academic setting. | “Students who generally are dysregulated, students that usually come with a label or a stigma against them, it gives them an opportunity to be seen in a different light…it just gives them an opportunity to be seen differently and then they find value in themselves.” T10, MS | Cognitive/  Emotional |
| Learning better | Students learn more effectively through active, hands-on, and/or outdoor ASK lessons. | “Yeah, I had a parent, she literally said to me that [her son] loves that you're doing ASK lessons because he finds it easier to learn.” T5, ES | Cognitive |
| Cognitive refresh or wake up | Becoming more focused or engaged, whether it is through waking students up or blowing off steam. | “I do think it helps them cognitively, though I can honestly say that when I do the active ASK activities in the morning…I find when they come in…they're just half asleep still. So, if I do the multiplication activity with them…it wakes them up, it peps them up.” T4, MS | Cognitive |
| Fewer interruptions | ASK lessons allow for fewer interruptions to learning both during the lessons and throughout the rest of the day. | “I find when they're not doing the ASK activities, there's more interruptions, whether it's interrupting myself or interrupting their peers because they want to have that social interaction, versus when we're doing ASK activities it's built in that they're supposed to talk to everybody and interact with everybody.” T9, MS | Cognitive |
| More focused after ASK | Students are focused, calm, and ready to learn when they are back in the classroom after an ASK lesson. | “So, if I do these kinds of lessons…students are more able to concentrate when they're in class. They're more focused when they get back in. They're ready to learn.” T1, ES | Cognitive |
| Not focused or on-task during lessons | Some teachers described struggling to keep students on-task during lessons. | “And I find the biggest challenge of all is just trying to keep them on task. I find sometimes when you take them outside, they can be really unfocused, not always, but sometimes. It really just depends on the day and the activity.” T2, ES | Cognitive (-ve) |
| Hard to focus after | After an ASK lesson the students sometimes have a hard time focusing with so much energy. | “I find for after we finish, it takes me a couple minutes to get them settled again, because they've been up and they've been, you know, sometimes like boisterous or yelling or, like they're doing whatever they're competitive.” T4, MS | Cognitive (-ve) |
| Theme 5: Pedagogical approaches as catalysts for wellbeing | | | |
| This theme captures how, alongside describing their perceived student experiences, teachers reflected on how those aspects of wellbeing emerged. | | | |
| Intentionally adding movement | Deliberate choices by teachers to incorporate movement into learning. | “So, I think of the game in the centre, putting everything there and having them move in so intentionally, I got that from ASK, I guess. I did have them working around the room and doing different things but now I guess after the training it's just more intentional.” T10, MS | N/A |
| Peer learning | Students learn through their peers during ASK lessons. | “I feel that they learn better when they're up and moving around and learning from each other instead of always listening to me trying to teach them a concept or review a concept with them.” T2, ES | N/A |
| Thinking outside the box | ASK lessons require students to get outside of their comfort zone and think outside of the box. | “And then your top end students, the ones that are flying high and you know that they're doing well…a physical ASK activity isn't going to hurt them in any way. It actually might change their thinking and make them do something a little bit differently because now they have to rely on a group member. So you're also challenging them in a different way. They might struggle with it in the beginning, but it's going to help them long term.” T9, MS | N/A |
| Adaptable to student needs | ASK is flexible and responsive to the diverse needs, abilities, and preferences of students. | “If there's children who struggle with the pen to paper and they're in a group, I can say, OK, I need to watch this kiddo today and see, ask questions, and then I do use it for them to show what they know. Sometimes. It depends on the child. There is a child that I based his entire report card off of situations like this because that's where he thrives.” T10, MS | N/A |
| Low pressure learning | ASK as a way to reinforce and practice previously taught skills in a low-pressure environment. | “I like how easy it is to assist students using it and how it reinforces learning in a positive, happy way for them.” T1, ES | N/A |
| Outdoor lessons | Being outdoors for the lessons often spiked even greater perceived benefits for students. | “And I think just being outdoors, I think that that's because you're not confined to a building. I think they just really like being outdoors. It almost feels more fun to them, if that makes sense.” T6, ES | N/A |
| Theme 6: ‘Running’ out of resources | | | |
| This theme describes the challenges teachers and their schools face in lacking resources and support to enable them to conduct ASK lessons, including lacking space and equipment, especially when the weather is not optimal to go outside, and lacking planning time and support, particularly in the context of large class sizes and students with disabilities. | | | |
| Lack of equipment | Teachers not having access to ASK materials, durable materials, or not having enough for the whole school. | “So, if I'm going to use [ASK materials] … I usually go first thing in the morning or right before recess and claim it, or even like the day before if I know I'm going to use it multiple times, I get it just before someone else uses it.” T2, ES | N/A |
| Lack of space | Spatial constraints due to physically small spaces, overcrowding, limited access to alternate spaces, overlapping class use of spaces, and weather conditions not allowing the use of the outdoors. | “That's a problem doing the ASK stuff. It's a small school…We're basically one hallway with three floors, but we're basically all the grades are in one hallway. It's an older school. The classrooms are an OK size, nothing for an ASK program though that you could do, which runs into a problem…and in the bad weather season like the winter when it's cold, that's a major challenge because I can't do it in my classroom. It takes a major upsetting of moving chairs and desks, and I can only do it with certain classes because of gym availability.” T3, MS | N/A |
| Maintaining and buying equipment | The equipment and materials used for ASK lessons can deteriorate and have to be maintained and this can fall on teachers to spend their own money replacing them or buying them to begin with. | “Proper ASK materials. Like I said, our school's pretty good to have those supplies and we kind of have a communal area in the staff room that we can grab the supplies, but those things cost money and the maintenance of them, teachers spend a lot of their own money in classrooms, and like everything costs a lot of money, and I've got two small kids of my own, so I've kind of reeled back on my own spending in my classroom. So just taking care of stuff and making sure that we have those things to properly execute those lessons.” T5, ES | N/A |
| Not having proper gear | Students do not have the proper outdoor gear for an outdoor ASK lesson. | “But that was a big problem that sometimes students arrive [but] they haven't got the right boots or jackets, but I was still going out because I told them that would happen. So pretty soon after their feet are cold or they’re cold, they remember the jackets the next day.” T1, ES | N/A |
| Planning time | The time demands to prepare an ASK lesson, through adapting curriculum to be active, competing demands, and gathering/making materials. But sometimes planning can be efficient through the use of simple worksheets made into active review lessons. | “So anytime a colleague is out, we have to cover the classes during our prep, which cuts into my prep time for ASK or doing anything else. So, time, finding time to do it on your prep, and that's the biggest obstacle. It's even more than doing the inside or outside…it's finding the time to come up with those activities and trying to decide what materials you need.” T3, MS | N/A |
| Competing demands | There are other events or teaching practices that sometimes take precedence over ASK lessons. | “It is important, but sometimes you just can't fit it in the schedule and like I hate to say it, but it's one of those things that get bumped, especially this time of year. The grade fives in my class they’re going to middle school so there's so many end-of-year things and it it's hard to get it in.” T5, ES | N/A |
| Database | Teachers appreciate having the ASK lesson database, especially visual examples of lessons, but some find it not as intuitive or user friendly as it could be. | “Some of the [lesson] descriptions [on the database] are a little brief and it would be nice to have like a visual somehow of like what it would look like. It's just words and [for] someone who's a visual learner. And teachers are tired at the end of the day, it would be nice to have it laid out a little bit different. And have…work pages there…to print off. Or to have something…you could edit it to make it your own.” T5, ES | N/A |
| Season or weather | Cold temperatures, snow, rain, mud, and ice restrict teachers’ ability to use outdoor spaces safely and consistently for ASK lessons. | “We did one [lesson] in the library, mostly in their classrooms, just because the landscape of the class outside school…it wasn't safe in certain places with the ice, so it wasn't that we were kind of afraid of the weather…But it was more the ice and the climate, like the weather conditions that kept us in.” T7, ES | N/A |
| Student Disabilities | Teachers are committed to inclusion but there are practical challenges and adaptations required for students with disabilities to participate in ASK lessons. | “I have a student who will be coming into my class in the next few years who is in a wheelchair... while the [outdoor learning area] is accessible to a wheelchair on the main path, anything off the path [where I usually set up] is not accessible...it’s so uneven terrain you can’t get wheelchairs in there.” T8, MS | N/A |
| Theme 7: Navigating the status quo | | | |
| The final theme explores the challenges associated with going against traditional learning ideas and pressures, whether through system-level standards, other teachers’ perspectives, or student dynamics and mindsets. | | | |
| New mindset or thinking | ASK takes a shift of mindset for both teachers and students. | “Some of the barriers might just be the students themselves getting used to it. I can think of it now being relatively easy cause I've done it for the last two years and a couple months. The beginning, you know, students were not as willing to accept that this was a way of learning.” T9, MS | N/A |
| Only teacher doing ASK in school | The only teacher who conducts ASK lessons at their school. | “I am sadly the only teacher here that has done anything with the ASK training too, I believe, there's no other teachers doing it.” T3, MS | N/A |
| Other teachers' perspectives | Other teachers in the school not on the same page in terms of the idea/benefits of ASK. | “Oh, you know, some barriers might be other teachers, just because they see a younger teacher doing something that's not in the classroom that doesn't look like [students are] learning. And I know people are stressing to make sure we cover this material in this amount of time and we can't do all this free play. Well, it's technically not free play. It's learning. And just getting some people to get over that can be a barrier.” T9, MS | N/A |
| Assessing outcomes | Teachers sometimes find it hard to assess students’ learning outcomes as ASK is less structured and product-based than the traditional learning assessments. | “You can't really assess it, like you know you can with a test, you know, what did you get a grade on with this? How are you doing? It's harder to assess them academically. But a lot of times I'll throw in something like an ASK activity, and I can see who's doing what. So…you can make it part of their mark a little bit, it's fun and it can be part of their grade and their participation.” T4, MS | N/A |
| Curriculum pressures | Teachers described significant pressure to meet curriculum requirements, making it challenging to find time for ASK unless they were clearly linked to curriculum outcomes. | “In terms of getting my curriculum done, I have to get so much done in the year. So, it can be challenging to lose 30 minutes.” T4, MS | N/A |
| Distracting other classes | Other classes are distracted by their students doing ASK lessons outside or in the hallway. | ‘This class is a little louder and a little noisier and a little more disruptive. And there's so many of them that it's hard to be in a hallway with other classes going on.” T6, ES | N/A |
| Student dynamics | Student engagement in ASK varied based on individual and group dynamics, with challenges including resistance to change, competitiveness, group conflicts, and differing levels of willingness to participate. | “It depends on the team. Some of the teams may not work well. I had a team recently where one of my kids said something very negative to another kid on his team. So, it depends on the kids, and it depends on the team. But those are also the things you have to work through, right?” T6, ES | N/A |
| Student safety | Teachers are thinking about how to keep students safe while doing an ASK lesson. | “Like [figuring out] how to create a learning environment and set expectations that are safe, respectful, and productive learning environments.” T7, ES | N/A |
| Teacher characteristics | Some teachers don’t enjoy or feel comfortable taking the students outside the classroom. | “But it depends on the teacher and if they like to go outside, [which is a] huge part of it.” T1, ES | N/A |

T: Teacher; ES: Elementary School; MS: Middle School; -ve: Negative
